# The lifetime of the oxygen‐evolving complex subunit PSBO depends on light intensity and carbon availability in *Chlamydomonas*

**DOI:** 10.1111/pce.14481

**Published:** 2022-11-17

**Authors:** André Vidal‐Meireles, Soujanya Kuntam, Eszter Széles, Dávid Tóth, Juliane Neupert, Ralph Bock, Szilvia Z. Tóth

**Affiliations:** ^1^ Laboratory for Molecular Photobioenergetics, Biological Research Centre Institute of Plant Biology Szeged Hungary; ^2^ Doctoral School of Biology University of Szeged Szeged Hungary; ^3^ Max Planck Institute of Molecular Plant Physiology Potsdam‐Golm Germany; ^4^ Present address: Institute of Plant Biology and Biotechnology (IBBP), Westfälische Wilhelms‐Universität Münster (WWU) Münster Germany

**Keywords:** CO_2_ availability, inducible amiRNA, photoinhibition, photosystem II, protein lifetime

## Abstract

PSBO is essential for the assembly of the oxygen‐evolving complex in plants and green algae. Despite its importance, we lack essential information on its lifetime and how it depends on the environmental conditions. We have generated nitrate‐inducible *PSBO* amiRNA lines in the green alga *Chlamydomonas reinhardtii*. Transgenic strains grew normally under non‐inducing conditions, and their photosynthetic performance was comparable to the control strain. Upon induction of the *PSBO* amiRNA constructs, cell division halted. In acetate‐containing medium, cellular PSBO protein levels decreased by 60% within 24 h in the dark, by 75% in moderate light, and in high light, the protein completely degraded. Consequently, the photosynthetic apparatus became strongly damaged, probably due to ‘donor‐side‐induced photoinhibition’, and cellular ultrastructure was also severely affected. However, in the absence of acetate during induction, PSBO was remarkably stable at all light intensities and less substantial changes occurred in photosynthesis. Our results demonstrate that the lifetime of PSBO strongly depends on the light intensity and carbon availability, and thus, on the metabolic status of the cells. We also confirm that PSBO is required for photosystem II stability in *C. reinhardtii* and demonstrate that its specific loss also entails substantial changes in cell morphology and cell cycle.

## Summary statement

The lifetime of the oxygen‐evolving complex subunit PSBO is diminished in high light and upon ample carbon supply in *Chlamydomonas*. PSBO is required for photosystem II stability, and its specific loss entails substantial changes in cell morphology and cell cycle.

## 1 INTRODUCTION

Photosystem II (PSII) carries out light‐energy conversion reactions. Its manganese cluster (Mn_4_CaO_5_) splits two water molecules into one molecule of oxygen and four protons in a light‐driven cycle. In plants and green algae, the Mn‐cluster is shielded on the thylakoid luminal side by the extrinsic proteins PSBO, PSBP, PSBQ, with apparent molecular masses of 33, 23 and 17 kDa, respectively (reviewed by Barber, 2016; Ifuku & Noguchi, 2016; Roose et al., 2016; Shen, 2015). These proteins stabilize the Mn‐cluster, and they probably regulate the access of Ca^2+^ and Cl^−^ and the retention of these ions to optimize oxygen evolution (Loll et al., 2005; Vinyard & Brudvig, 2017). In addition, the extrinsic subunits protect the Mn‐cluster from reductants (Bricker et al., 2012; Ghanotakis et al., 1984; Popelkova et al., 2011), including luminal ascorbate (Podmaniczki et al., 2021). The extrinsic proteins, PSBO in particular, may also regulate water access and proton removal from the Mn‐cluster via a hydrogen‐bonding network (Guskov et al., 2009; Ho & Styring, 2008; Offenbacher et al., 2013). In addition, PSBO has also been suggested to exhibit carbonic anhydrase activity (Lu & Stemler, 2002).

In line with its crucial role in photosynthetic water splitting, PSBO is essential for photoautotrophic growth in vascular plants and algae (Liu et al., 2009; Mayfield et al., 1987; Pigolev & Klimov, 2015; Yi et al., 2005). In *Arabidopsis thaliana*, PSBO has two isoforms, PSBO1 and PSBO2 that are encoded by *PSBO1* (At5g66570) and *PSBO2* (At3g50820). T‐DNA knockout mutants of *PSBO1* (*psbo1*) exhibit retarded growth, malfunction of both the donor and acceptor sides of PSII, and the mutants are highly susceptible to photoinhibition. The absence of *PSBO2* hardly affects PSII activity and plant growth (Allahverdiyeva et al., 2009; Lundin et al., 2007); instead, the PSBO2 protein may act as a GTPase, regulating PSII repair in *Arabidopsis* (Lundin et al., 2007; Spetea et al., 2004). In *Chlamydomonas reinhardtii*, PSBO is encoded by a single gene, *Cre09.g396213*, and it is indispensable for oxygen evolution (Mayfield et al., 1987; Pigolev & Klimov, 2015).

The subunit composition of the photosynthetic machinery exhibits some flexibility. Response to environmental perturbations and the steady‐state maintenance of the photosynthetic machinery requires that the changes take place on a relatively short timescale (Nelson et al., 2014). It is well known that the PsbA subunit of PSII exhibits a particularly fast turnover (in the time range of a few hours) to mitigate photodamage, an inherent accompanying event of photosynthesis. The half‐life of PsbA is inversely correlated to light intensity (e.g., Schuster et al., 2020), and certain environmental stress conditions also enhance its degradation (Aro et al., 1993; Marutani et al., 2012; Mittal et al., 2012). In vascular plants, the other PSII subunits have been shown to have a rather slow turnover (L. Li et al., 2017).

PSBO is a relatively thermostable protein (Lydakis‐Simantiris et al., 1999) and has been suggested to have a long lifetime, even in its free form (Hashimoto et al., 1996). In Synechocystis grown at very low light, PSBO has a half‐life of 24−33 h (Yao et al., 2012). On the other hand, it was shown in isolated PSII membranes that PSBO might become oxidatively damaged under light stress, and its binding capacity to PSII may be reduced (Henmi et al., 2004). It was also shown that PSBO could be degraded in a redox (thioredoxin)‐dependent manner in both higher plants and cyanobacteria, and its assembly into PSII protects it from proteolytic degradation (Roberts et al., 2012).

Microalgae are thought to adjust the concentration of photosynthetic proteins to match the environmental conditions through cell division in a way that cell division is coupled with the tuning of gene expression, leading to an adjustment of the photosynthetic complex stoichiometry (Davis et al., 2013; Meagher et al., 2021). However, this concept does not explain how algae can cope with varying light conditions under nutrient‐limiting conditions when cell division is limited.

In this work, we aimed at investigating the dependence of the lifetime of PSBO on environmental conditions in the model organism *C. reinhardtii*. To this end, we have generated nitrate‐inducible artificial microRNA (amiRNA) lines targeting *PSBO*. The advantage of the amiRNA approach is that off‐target effects and silencing are minimized relative to antisense and inverted repeat constructs (Molnar et al., 2009), and the inducible system enables normal growth and development under non‐inducing conditions (Schmollinger et al., 2010).

By inducing the *PSBO* amiRNA construct, we confirmed that PSBO is indispensable for the stability of mature PSII reaction centres. In addition, we found that the time course of diminishment of PSBO protein level (i.e., its lifetime) is strongly dependent on light intensity and carbon availability, and thus, on the metabolic status of the cell.

## 2 MATERIALS AND METHODS

### 2.1 Algal strains and growth conditions

*C. reinhardtii* strain cw15‐325 (cw_d_mt^+^arg7 nit1^+^ nit2^+^) was provided by Dr. Michael Schroda (Technische Universität Kaiserslautern). Strain cw15‐325 was used as the recipient strain for transformation with the nitrate‐inducible *PSBO* amiRNA and control constructs (see below). Algal strains were grown mixotrophically in Tris‐acetate‐phosphate (TAP) medium on a rotatory shaker at 22°C and 80 µmole photons m^−2^ s^−1^ of continuous illumination when required. The cw15‐325 strain is auxotrophic for arginine, therefore its TAP medium was supplemented with 100 µg/ml of l‐arginine.

### 2.2 Nitrate‐inducible PSBO amiRNA design and transformation

The amiRNAs targeting the *C. reinhardtii PSBO* locus were designed according to the instructions by Molnar et al. (2009) using the WMD2 tool at http://wmd2.weigelworld.org (Ossowski et al., 2008). One amiRNA targeting the coding region (CDS, denoted as *PSBO‐A* strains), and one targeting the 3'UTR of *PSBO* (denoted as *PSBO‐B* strains) were selected. The constructs contained one mismatch at position 5 relative to their amiRNA* sequences (Supporting Information: Table S1). The synthesized sense and antisense oligonucleotides with SpeI‐compatible ends were annealed by boiling and slowly cooling down and ligated into the SpeI‐digested *pMS539* vector. Its *NIT1* promoter drives the expression of the *PSBO* amiRNA construct, upon changing the nitrogen source from ammonium to nitrate in the growth medium (Schmollinger et al., 2010). Screening for correct clones was done as described by Molnar et al. (2009) and Schmollinger et al. (2010). The plasmid was linearized by digestion with HindIII and transformed into cw15‐325 by vortexing with glass beads (Kindle, 1990).

### 2.3 Induction of the amiRNA constructs, light, colchicine and CO_2_ treatments

Precultures were grown in 50 ml Erlenmeyer flasks for 3 days in TAP medium at 22°C and 80 µmole photons m^−2^ s,^−1^ corresponding to Time 0 of the treatments. For the induction of the *PSBO* amiRNA construct, cells were washed four times with nitrogen‐free TP medium and transferred to nitrate‐containing (7.48 mM) TAP or TP medium in 250 ml Erlenmeyer flasks. The chlorophyll (Chl) content was set to 5 µg Chl (a + b)/ml, determined according to Porra et al. (1989). At the start of the induction, the cultures were placed either in the dark, under continuous normal light (NL, 100 µmole photon m^−2^ s,^−1^ using Sylvania luxline cool white 4000K fluorescent tube), or in continuous high light (HL, 530 µmole photon m^−2^ s,^−1^ using cool white 4000K LED) for 4 days. The growth and photosynthetic parameters of *C. reinhardtii* are very similar under these two light sources at identical intensities (Bialevich et al., 2022).

For colchicine treatment, colchicine (5 µM; Duchefa Biochemie) was added to the cultures after washing the cells with nitrogen‐free medium and setting the Chl (a + b) content to 5 µg Chl (a + b)/ml in nitrate‐containing TAP or TP medium.

To investigate the effects of CO_2_‐supplementation, a Multi‐Cultivator MC 1000‐OD instrument (Photon Systems Instruments) was used. At the start of cultivation, the cells were transferred to nitrate‐containing TP medium and the Chl (a + b) content was set to 5 µg Chl (a + b)/ml. The cultures were cultivated at 22°C, 100 µmole photons m^−2^ s^−1^ in continuous light without or with CO_2_‐bubbling (1%) for 4 days.

### 2.4 Analyses of gene expressions

For isolation of RNA, 2 ml of culture was harvested at each time‐point, and the Direct‐Zol RNA kit was used, following the recommendation of the manufacturer's (ZymoResearch). To remove contaminating DNA from the samples, the isolated RNA was treated with DNaseI (ZymoResearch). Small RNAs were isolated as described by Molnár et al. (2007). RNA integrity was checked on a 1% (w/v) denaturing agarose gel.

The primers used for real‐time qPCR analysis (Supporting Information: Table S1) of *CDKG1*, *CYCA1*, *WEE1* and *POLA4* were published earlier by Bisova et al. (2005). The primer pairs for the reference genes (*bTub2*, *actin*, *UBQ*) were published in Vidal‐Meireles et al. (2017); the primer pair for the small RNA reference gene (*U4 snoRNA*) was published earlier by H. Li et al. (2018).

For total RNA, reverse transcription was primed with random hexamers using 1 µg of total RNA and FIREScript RT kit (Solis Biodyne). For small RNAs, reverse transcription after polyadenylation was performed as in Varkonyi‐Gasic et al. (2007). Briefly, small RNAs were polyadenylated with two units of Poly(A) Polymerase (Thermo Fisher Scientific) in a 25 µl reaction mix including 500 ng RNA, 5 µl of 5 × E‐PAP reaction buffer, 0.3 µl of 100 mM ATP and 2.5 µl of 25 mM MgCl_2_ at 37°C for 1 h, before performing the reverse transcription by adding to the polyadenylation reaction mix the FIREScript RT kit (Solis Biodyne) and 1 µl of 1 µM RT primers for pulsed PCR incubation (30 min at 16°C, followed by 60 cycles of 30 s at 30°C, 30 s at 42°C and 1 s at 50°C) before inactivating the enzyme (5 min at 85°C). To confirm the absence of DNA contaminations, an aliquot of the RNA sample was used without reverse transcriptase.

Real‐time qPCR analysis was performed using a Bio‐Rad CFX384 Touch Real‐Time PCR Detection System using HOT FIREPol EvaGreen qPCR Mix Plus (Solis Biodyne) for cDNA detection, or LightCycler TaqMan Master supplemented with the Universal Taqman probe #21 (Roche Molecular Systems) for amiRNA detection. The data are presented as fold‐change in mRNA transcript abundance, normalized to the average of the reference genes, and relative to the *EV31* control sample or to the 0 h time point (for amiRNA detection). Real‐time qPCR analysis was carried out with three technical replicates for each sample and three to five biological replicates were analysed; the standard error (SE) was calculated based on the different transcript abundances amongst the independent biological replicates.

### 2.5 Determination of cell size and cell density

The cell density and cell size were determined either by a Millipore Scepter™ 2.0 hand‐held cell counter (Merck KGaA) or Luna‐FL™ dual fluorescence cell counter (Logos Biosystems Inc.).

### 2.6 Oxygen evolution measurements

An oxygen optode with a 4 mm outside diameter (Fibox 3; Presens GmbH) was used to measure the dissolved oxygen content in the alga samples. 1.9 ml of culture was collected at each time‐point, supplemented with 50 mM sodium bicarbonate, and continuously stirred with a magnetic stir bar in darkness. After 60 s of dark incubation, when a stable baseline was reached, the sample was illuminated with continuous white light (about 800 µmol photons m^−2^ s^−1^) provided by a fibre optic light source (Schott KL 1500; Schott AG). The dissolved oxygen concentration was measured continuously by the manufacturer's software (OxyView ‐ PST3‐V5.32 02/2004; PreSens GmbH) for 10 min, at a sampling interval of 1 s. The oxygen sensor was calibrated with two points, that is, air‐saturated water ‘Cal 100’ (100% air saturation) and anoxic water ‘Cal 0’ (1 g of Na_2_SO_3_ in 100 ml distilled water) before the measurement. The oxygen evolution rate was determined from the slope of the linear fitting of the original traces during the illumination phase and expressed in μmol O_2_/h/million cells units.

### 2.7 Chl a fluorescence measurements

Chl *a* fluorescence measurements were carried out as described in Nagy et al. (2018). Briefly, *C. reinhardtii* cultures were dark‐adapted for about 15 min, and then 3 ml of cell suspension was filtered onto a Whatman glass microfibre filter (GF/B) and measured with a HandyPEA instrument (Hansatech Instruments Ltd).

### 2.8 Immunoblot analysis

At each sampling point, 2 ml of culture were collected, spun down for the removal of the supernatant, and frozen in liquid nitrogen. The samples were then solubilized as described in Nagy et al. (2018). Extracts originating from equal amounts of cells were loaded for each sample to account for the differences in growth rates between the strains and conditions. That is, cell extract equivalent to 1 million cells was mixed with 6x Laemmli buffer (375 mM Tris/HCl [pH 6.8], 60% [v/v] glycerin, 12.6% [w/v] sodium dodecyl sulfate, 600 mM dithiothreitol, 0.09% [w/v] bromophenol blue) and incubated at 75°C for 10 min before loading. Protein separation and western blot were carried out as described in Podmaniczki et al. (2021). Specific polyclonal antibodies (produced in rabbit) against PSBO, PsbA (N‐terminal), CP47, PSBP, PetA, PetB, PsaA and RbcL were purchased from Agrisera AB.

### 2.9 Confocal microscopy

Imaging was performed using an Olympus Fluoview FV1000 microscope (Olympus Life Science Europa GmbH). Samples were prepared for imaging on clean glass coverslips (Menzel‐Gläser, 24 × 40 mm; Thermo Fisher Scientific), and wiped with 100% ethanol. The cells were immobilized on the coverslips under 0.4% agarose slab of 0.25 × 0.5 × 0.5 cm. Single optical sections or successive 3D optical sections of the cells were taken using the UPLSAPO ×60 (NA: 1.35) oil immersion objective. Microscope configuration was as follows: sampling speed: 4 μs/pixel; line averaging: ×3; scanning mode: unidirectional; zoom: ×7; excitation: 488 nm (Nile Red), 543 nm (Chl *a* fluorescence); maximum laser transmissivity value: 10% (488 nm), 20% (543 nm). Nile Red fluorescence and Chl autofluorescence was detected between 550 and 600 nm and 650−750 nm, respectively. Images were pseudocolored and analysed using Olympus Fluoview software (version 4.0.3.4) and ImageJ (version 1.52a). Representative images out of ~100 observed cells are presented.

### 2.10 Statistical analysis

The presented data are based on at least three independent experiments. The exact number of the biological repetitions is indicated in the figure captions. When applicable, averages and +SE were calculated. The significance of the mean differences between the mutants and the *EV31* strain under each growth condition were analysed by Student's *t*‐test using GraphPad Prism5 software, and the significance levels are presented when applicable.

## 3 RESULTS

### 3.1 Generation of nitrate‐inducible amiRNA lines targeting PSBO in *C. reinhardtii*

The *pMS539* vector contains the *ARG7* gene as a selection marker and the *NIT1* promoter to drive the expression of amiRNA upon the transfer of cells from ammonium‐containing to nitrate‐containing growth medium (Schmollinger et al., 2010). This approach enables relatively normal growth of the transformants under non‐inducing conditions, even when targeting genes essential for cell survival.

**Figure 1:**
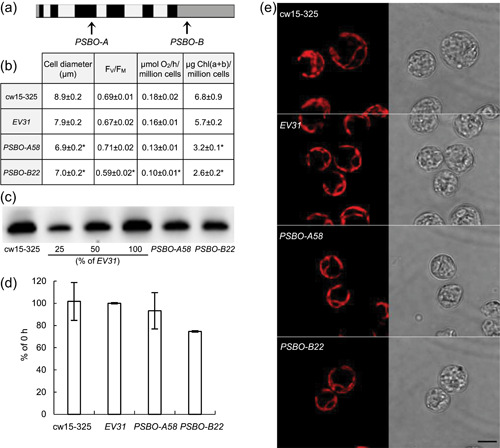
Generation and characterization of nitrate‐inducible *PSBO amiRNA* transformants under non‐inducing (i.e., control) conditions in TAP medium at normal light (100 µmol photons m^−2^ s^−1^). (a) Physical map of the *PSBO* gene (obtained from Phytozome v12.1.6) with the two *PSBO* amiRNA target sites (PSBO‐A and PSBO‐B, indicated by arrows). Exons are shown in black, introns in light grey, and promoter, 5'UTR and terminator sequences in the dark grey. (b) Cell diameter, F_V_/F_M_ value, O_2_ evolution rate and Chl content of the arginine‐supplemented cw15‐325 parent strain, an empty vector control strain (*EV31*), and two selected *PSBO amiRNA* transformants, targeting the coding region (*PSBO‐A58*) or the 3'UTR (*PSBO‐B22*) of *PSBO*. (c) Representative example of immunoblot analysis and densitometry of three replicates (d) to relatively quantify the amount of PSBO in the control strains and the PSBO amiRNA transformants. The samples were loaded on equal cell numbers. (e) Chl autofluorescence and their corresponding transmission images of the control strains and the *PSBO amiRNA* transformants. Scale bar: 5 µm. Values are means ± SE of three biological replicates. Statistical significance levels are presented relative to the *EV31* strain as: *p < 0.05. amiRNA, artificial microRNA; Chl, chlorophyll; SE, standard errors; TAP, Tris‐acetate‐phosphate. [Color figure can be viewed at wileyonlinelibrary.com]

We transformed the cell wall‐deﬁcient strain cw15‐325 carrying a mutation in argininosuccinate lyase (ARG7) with the *PSBO*‐speciﬁc nitrate‐inducible amiRNA vectors targeting the coding region (CDS) or the 3'UTR of *PSBO* mRNA. In the case of the CDS‐targeting amiRNA (*PSBO‐A*) construct (Figure 1a), 126 arginine‐prototrophic colonies were tested by PCR, and 89 of them were found to carry the whole amiRNA cassette (Supporting Information: Table 1). For the PSBO 3'UTR‐targeting amiRNA (*PSBO‐B*) construct (Figure 1a), 61 of the 96 tested arginine‐prototrophic colonies carried the amiRNA cassette. Empty vector (EV) transformants were used as control strains. In preliminary experiments, 10 lines were tested for the *PSBO‐A* and *PSBO‐B* constructs, of which 80%−90% bleached within 2 days. Based on this, we have selected one inducible amiRNA transformant targeting the CDS of *PSBO* (*PSBO‐A58*), and one targeting the 3'UTR of *PSBO* (*PSBO‐B22*). Thus, two entirely independent *PSBO* amiRNA constructs were used in the experiments, and we employed an EV strain (*EV31*) as a control.

Under non‐inducing conditions (i.e., when grown on ammonium), both *PSBO* amiRNA lines showed a moderate decrease in cell size, cellular Chl (a + b) content, oxygen evolution and one of the lines, *PSBO‐B22*, also had a slightly diminished F_V_/F_M_ value (Figure 1b). In the *PSBO‐A58* and *PSBO‐B22* lines, the amount of PSBO was slightly diminished relative to the control strains cw15‐325 and *EV31* under non‐inducing conditions (Figure 1c,d). The *PSBO* amiRNA transformants had normal cell and chloroplast morphology under non‐inducing conditions (Figure 1e). Our data indicate that the *NIT1* promoter is somewhat leaky, in agreement with Schroda (2019) and Theis et al. (2020). Nevertheless, these changes were relatively mild. Thus, the inducible *PSBO* amiRNA transformants were suitable for further experiments.

### 3.2 The diminishment of PSBO protein level is enhanced in the presence of acetate

**Figure 2:**
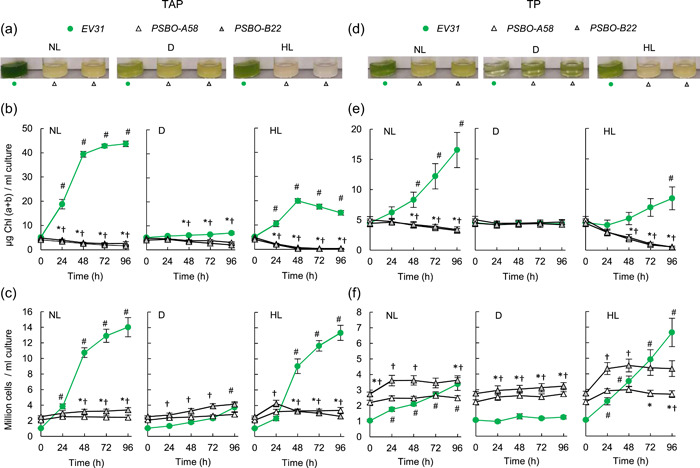
The effects of downregulating *PSBO* via the nitrate‐inducible amiRNA approach in the presence (TAP medium) or absence of acetate (TP medium) at normal light (100 µmol photons m^−2^ s^−1^, NL), in the dark (D), and at high light (530 µmol photons m^−2^ s^−1^, HL). (a) Photographs of the *EV31* control strain and *PSBO‐A58* and *PSBO‐B22 PSBO* amiRNA transformants 48 h after placing them in inducing conditions in TAP medium. (b) Changes in Chl (a + b) content following induction in TAP medium. (c) Changes in cell density following induction in TAP medium. (d) Photographs of the *EV31* control strain and *PSBO‐A58* and *PSBO‐B22 PSBO* amiRNA transformants 48 h after placing them in inducing conditions in TP medium. (e) Changes in Chl (a + b) content following induction in TP medium. (f) Changes in cell density following induction in TP medium. Values are means ± SE of four biological replicates. Statistical significance levels are presented relative to the *EV31* strain (at each individual time‐point) as *p* < 0.05, *PSBO‐A58, †*PSBO‐B22*. For comparison with the (0 h) *EV31* sample, statistical significance levels are presented as #*p* < 0.05. amiRNA, artificial microRNA; Chl, chlorophyll; SE, standard errors; TAP, Tris‐acetate‐phosphate. [Color figure can be viewed at wileyonlinelibrary.com]

Upon transfer of the *PSBO* amiRNA lines to inducing conditions in the presence of acetate (TAP medium), the Chl (a + b) content of the cultures decreased. Under NL (100 µmol m^−2^ s^−1^), about 50% decrease in Chl (a + b) content was observed whereas, under HL (530 µmol photons m^−2^ s^−1^), complete bleaching of the cultures was observed within 48 h (Figure 2a,b). Interestingly, the *PSBO* amiRNA cultures bleached partially even in the dark (Figure 2a,b), and cell proliferation stopped both in the light and in the dark (Figure 2c). Accordingly, the cellular Chl (a + b) content was strongly diminished, which was most striking in the *PSBO* amiRNA cultures induced at HL (Supporting Information: Figure 1).

In the absence of acetate (TP medium) under NL and in the dark, the Chl (a + b) content decreased moderately (by about 15% in 48 h), and complete bleaching occurred under HL only after 96 h of induction of the *PSBO* amiRNA lines (Figure 2d,e and Supporting Information: Figure 1). Cell division of the *PSBO* amiRNA lines was largely prevented in TP medium as well both in light and dark conditions (Figure 2f).

Regarding the *EV31* control strain, we noted that cell division occurred in the nitrate‐containing TAP medium at NL at a rate similar to that of ammonium‐containing TAP medium and at similar light conditions, which is about three times a day (Puzanskiy et al., 2018; Zhang et al., 2020). In the dark, culture growth was slow even in the presence of acetate, in agreement with Juergens et al. (2016).

**Figure 3:**
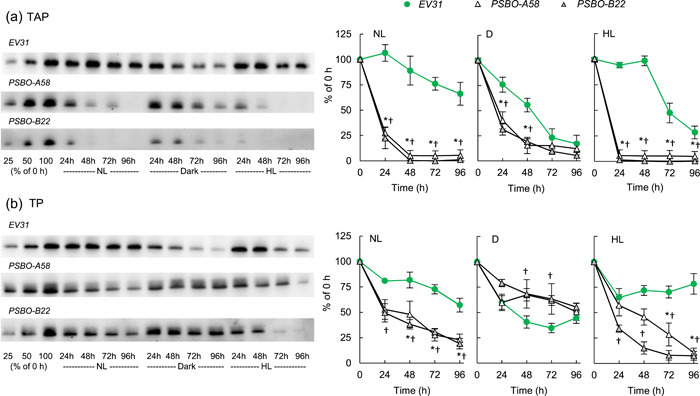
Changes in the relative amounts of PSBO under inducing conditions at normal light (100 µmol photons m^−2^ s^−1^, NL), in the dark (D), and at high light (530 µmol photons m^−2^ s^−1^, HL) in the empty vector *EV31* strain and the *PSBO‐A58* and *PSBO‐B22* nitrate‐inducible *PSBO*‐amiRNA transformants. (a) Representative immunoblots and densitometry analysis to monitor the loss of PSBO in the three genotypes in nitrate‐containing TAP medium. (b) Representative immunoblots and densitometry analysis to monitor the loss of PSBO in the three genotypes in nitrate‐containing TP medium (i.e., without acetate). The samples were loaded on equal cell numbers. Values are means ± SE of three biological replicates. Statistical significance levels are presented relative to the *EV31* strain as *p* < 0.05, *PSBO‐A58, †*PSBO‐B22*. amiRNA, artificial microRNA; SE, standard errors; TAP, Tris‐acetate‐phosphate. [Color figure can be viewed at wileyonlinelibrary.com]

Since cell division was halted upon the induction of the *PSBO* amiRNA constructs in all conditions, the diminishment of PSBO level on an equal cell number basis can be used to obtain information about the lifetime of PSBO. We observed that upon induction of the amiRNA construct in nitrate‐containing TAP medium, the cellular PSBO content rapidly diminished in the light: in NL, about 25% of PSBO remained in both *PSBO* amiRNA transformants after 24 h of induction, and by the 48th hour, it became almost entirely degraded (Figure 3a). Under HL, almost all PSBO was degraded within 24 h in both *PSBO* amiRNA strains. The rate of PSBO down‐regulation was lowest in the dark. We observed a slow diminishment of PSBO content in the *EV31* strain as well under all light conditions (Figure 3a), in the dark in particular, which is in agreement with the earlier findings of for example, Malnoë et al. (1988), that PSBO is present in lower amounts in the dark than in the light.

The cellular PSBO protein level decreased more slowly when the cultures were kept in acetate‐free medium (TP) without CO_2_ supplementation: at the 48 h time point, about 45% of PSBO was retained in both strains at NL, whereas at HL, 46% and 15% of PSBO was present in the *PSBO‐A58* and *PSBO‐B22* transformants, respectively (Figure 3b). In the dark, PSBO was relatively stable (about 50% diminishment occurred in 96 h) both in the *EV31* control and the nitrate‐induced *PSBO amiRNA* lines (Figure 3b).

**Figure 4:**
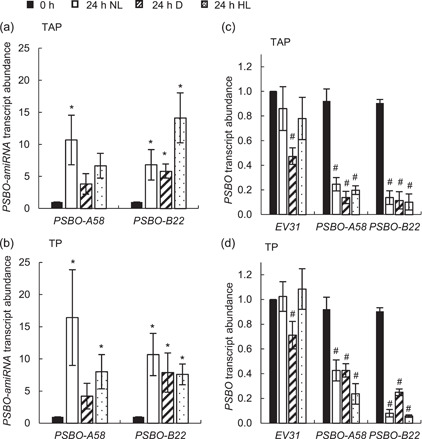
Determination of the relative transcript abundance of *PSBO* amiRNA and *PSBO* mRNA levels upon induction of the constructs with nitrate at normal light (100 µmol photons m^−2^ s^−1^, NL), in the dark (D), and at high light (530 µmol photons m^−2^ s^−1^, HL). (a) *PSBO* amiRNA levels in the *PSBO‐A58* and *PSBO‐B22* nitrate‐inducible *PSBO* amiRNA transformants in TAP medium. (b) *PSBO* amiRNA levels in TP medium. (c) *PSBO* mRNA levels in the *EV31* control strain and the *PSBO‐A58* and *PSBO‐B22 PSBO* amiRNA transformants in TAP medium. (d) *PSBO* mRNA levels in TP medium. Values are means ± SE of four biological replicates. Statistical significance levels are presented as *p* < 0.05, * versus 0 h, # versus *EV31* 0 h. amiRNA, artificial microRNA; SE, standard errors; TAP, Tris‐acetate‐phosphate.

To exclude the possibility that these differences are due to inefficient amiRNA induction under any of the tested conditions, we also determined the relative transcript abundance of *PSBO* amiRNA. Figure 4a,b shows that the construct was effectively induced upon transfer of the cultures from ammonium‐containing to nitrate‐containing medium in all conditions. Accordingly, the relative transcript abundance of *PSBO* was decreased by at least 60% in both *PSBO amiRNA* transformants in all inducing conditions (Figure 4c,d). In addition, it was shown earlier that in *C. reinhardtii*, amiRNA may act via translational arrest mechanism as well (Vidal‐Meireles et al., 2017), therefore the actual diminishment in the rate of PSBO synthesis could be even stronger as expected based on the *PSBO* transcript level. We noted that the *PSBO* transcript level significantly decreased in the dark in the *EV31* strain as well (Figure 4c,d), which is in line with the reduction of PSBO in the dark (Figure 3a,b).

### 3.3 Downregulating PSBO entails substantial cellular damage

**Figure 5:**
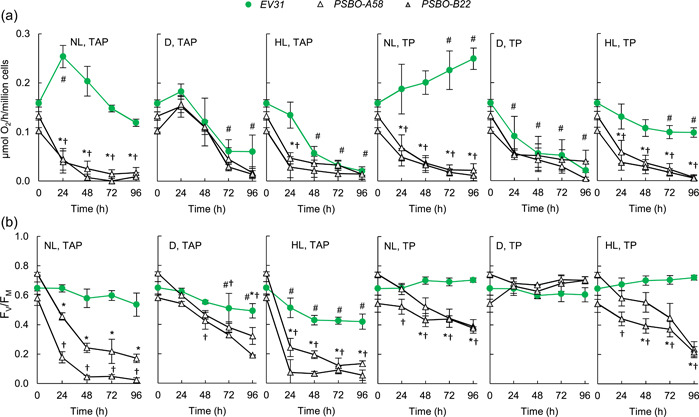
The effects of downregulating *PSBO* on photosynthesis via the nitrate‐inducible amiRNA approach in the presence (TAP medium) or absence of acetate (TP medium) at normal light (100 µmol photons m^−2^ s^−1^ NL), in the dark (D) and at high light (530 µmol photons m^−2^ s^−1^, HL). (a) Changes in O_2_ evolution. (b) Changes in photosynthetic efficiency as assessed by the F_V_/F_M_ Chl *a* fluorescence parameter. Values are means ± SE of four biological replicates. Statistical significance levels are presented relative to the *EV31* strain (at each individual time‐point) as *p* < 0.05, *PSBO‐A58, †*PSBO‐B22*. For comparison with the (0 h) *EV31* sample, statistical significance levels are presented as #*p* < 0.05. amiRNA, artificial microRNA; Chl, chlorophyll; SE, standard errors; TAP, Tris‐acetate‐phosphate. [Color figure can be viewed at wileyonlinelibrary.com]

Next, photosynthetic parameters were determined. The rate of oxygen evolution (Figure 5a) strongly decreased, in line with the loss of PSBO at NL and HL conditions in both *PSBO* amiRNA lines (cf. Figure 2). In addition, we noted a steady decrease in the rate of oxygen evolution in the dark in all strains, including the *EV31* strain (Figure 5a). The F_V_/F_M_ values also decreased, albeit less drastically than the rate of oxygen evolution (Figure 5b).

In TP medium, a similar tendency was observed in the rate of oxygen evolution, except that the decrease occurred more slowly (Figure 5a). However, the F_V_/F_M_ value remained relatively high in both *PSBO* amiRNA lines at NL, HL and in the dark (Figure 5b). The discrepancy between oxygen evolution rate and the F_V_/F_M_ values may be explained by the measurements of oxygen evolution having been performed at intense illumination lasting for several minutes, during which any reduction in oxygen evolution capacity can manifest itself. On the other hand, the F_V_/F_M_ value determination requires only a short illumination time (about 300 ms), and a single charge separation can lead to a sizeable F_V_/F_M_ value even in the complete absence of OEC activity (Tóth et al., 2005, 2009).

We also noted that the HL treatment in TP medium in the *EV31* strain caused only a moderate reduction of the oxygen evolving activity (Figure 5a), thus the treatment itself was not excessively damaging to the photosynthetic apparatus.

**Figure 6:**
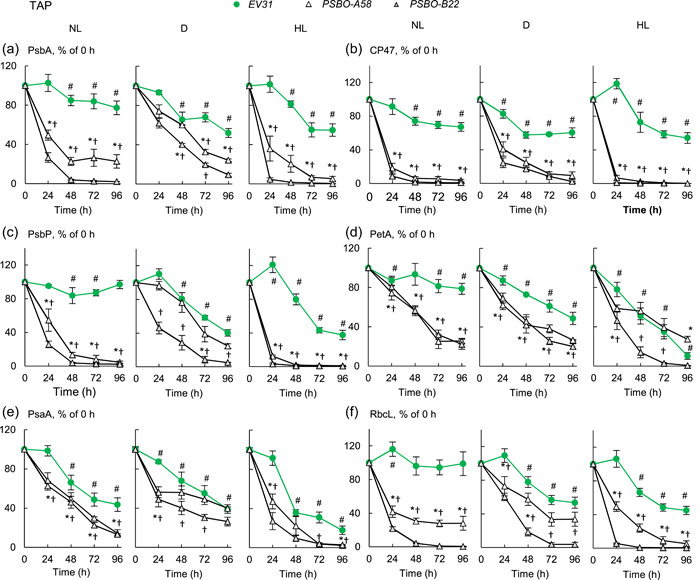
Densitometry analysis of immunoblots for the semiquantitative determination of selected photosynthetic subunits following downregulating *PSBO* via the nitrate‐inducible amiRNA approach in the presence of acetate (TAP medium) at normal light (100 µmol photons m^−2^ s^−1^ NL), in the dark (D) and at high light (530 µmol photons m^−2^ s^−1^, HL). (a) PsbA, (b) CP47, (c) PsbP, (d) PetA, (e) PsaA, (f) RbcL. Samples were loaded based on equal cell numbers. A dilution series (25%, 50% and 100%) of the 0 h‐sample of each genotype was used as the reference for the densitometry analysis. Densitometry analysis was based on three independent experiments; representative blots are shown in Supporting Information: Figure 2. Statistical significance levels are presented relative to the *EV31* strain (at each individual time‐point) as *p* < 0.05, *PSBO‐A58, †*PSBO‐B22*. For comparison with the (0 h) *EV31* sample, statistical significance levels are presented as #*p* < 0.05. amiRNA, artificial microRNA; TAP, Tris‐acetate‐phosphate. [Color figure can be viewed at wileyonlinelibrary.com]

The induction of the *PSBO* amiRNA constructs in TAP medium at NL resulted in the losses of PsbA, CP47 and PSBP when assessed based on equal cell numbers (Figure 6); after 48 h at NL, about 0%−20% of these subunits remained and interestingly, CP47 was diminished most rapidly (Figure 6b). The amounts of PsbA, CP47 and PSBP decreased in the dark as well, albeit at a slower rate. The diminishment of PSII subunits was remarkably rapid at HL, where a quasi‐complete loss of these subunits occurred within 24 h (Figure 6a−c, typical blots are presented in Supporting Information: Figure 2). Upon induction of the *PSBO* amiRNA construct in NL, the levels of PetA (the cytochrome *f* subunit of the cytochrome *b*_*6*_ *f* complex), PsaA (a reaction centre protein of PSI) and RbcL (the large subunit of Rubisco) also decreased. Among these photosynthetic subunits, interestingly, the diminishment of RbcL was the fastest. At HL, the loss of PetA, PsaA and RbcL occurred more rapidly than at NL and slightly more slowly in the dark (Figure 6d−f, Supporting Information: Figure 2). We note that in the *EV31* strain, the amounts of these photosynthetic subunits also moderately decreased in the dark and in HL, which is in line with the partial loss of PSBO under these conditions (c.f. Figure 3).

**Figure 7:**
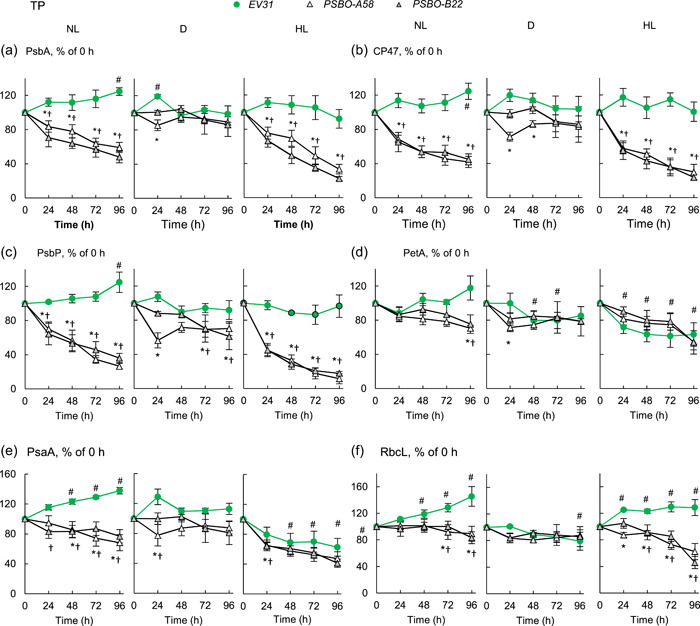
Densitometry analysis of immunoblots for the semiquantitative determination of certain photosynthetic subunits following downregulating *PSBO* via the nitrate‐inducible amiRNA approach in the absence of acetate (TP medium) at normal light (100 µmol photons m^−2^ s^−1^ NL), in the dark (D) and at high light (530 µmol photons m^−2^ s^−1^, HL). (a) PsbA, (b) CP47, (c) PsbP, (d) PetA, (e) PsaA, (f) RbcL. Samples were loaded based on equal cell numbers. A dilution series (25%, 50% and 100%) of the 0 h‐sample of each genotype was used as the reference for the densitometry analysis. Densitometry analysis was based on three independent experiments; representative blots are shown in Supporting Information: Figure 3. Statistical significance levels are presented relative to the *EV31* strain (at each individual time‐point) as *p* < 0.05, *PSBO‐A58, †*PSBO‐B22*. For comparison with the (0 h) *EV31* sample, statistical significance levels are presented as #*p* < 0.05. amiRNA, artificial microRNA. [Color figure can be viewed at wileyonlinelibrary.com]

In TP medium, the photosynthetic subunits were much more stable. In the *PSBO amiRNA* transformants, the PSII reaction centre subunits were largely preserved in the dark; their amounts decreased slowly in NL and somewhat faster in HL, and at least 30% of each subunit remained after 48 h of induction (Figure 7a−c). The other photosynthesis‐related proteins (PetA, PsaA and RbcL) were essentially retained in the *PSBO* amiRNA lines (Figure 7d−f, typical blots are presented in Supporting Information: Figure 3). In the *EV31* line, there was no significant decrease in the amounts of photosynthetic complexes in NL and D, whereas in HL, a moderate decrease of PetA and PsaA occurred (Figure 7a−f, Supporting Information: Figure 3).

**Figure 8:**
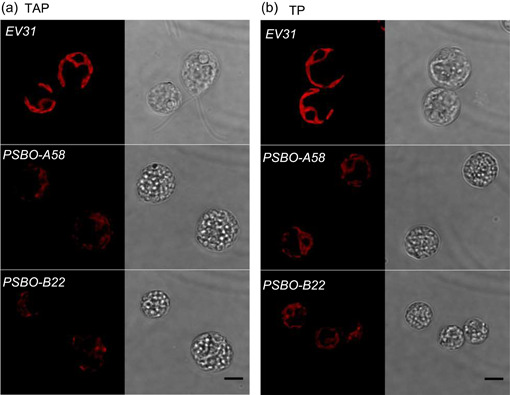
Confocal fluorescence microscope images at 48 h following downregulating PSBO via the nitrate‐inducible amiRNA approach at normal light (100 µmol photons m^−2^ s^−1^). Single plane chlorophyll autofluorescence (red) and their corresponding transmission images (black and white) are shown. (a) In the presence of acetate (TAP). (b) In the absence of acetate (TP). Scale bar: 5 μm. amiRNA, artificial microRNA; TAP, Tris‐acetate‐phosphate. [Color figure can be viewed at wileyonlinelibrary.com]

These experiments were complemented by confocal laser‐scanning microscopy. In TAP medium, upon loss of PSBO, the intensity of the Chl autofluorescence diminished (the 48 h time point at NL is presented in Figure 8a; HL and D treatments shown in Supporting Information: Figures 4 and 5), and chloroplast ultrastructure was severely affected (Figure 8a, Supporting Information: Figures 4 and 5). Transmission images revealed the increased formation of large globular structures in the *PSBO* amiRNA transformants, which were suspected to be lipid droplets (Terashima et al., 2015). Nile Red staining confirmed the presence of lipid droplets (Supporting Information: Figure 6). They were detected in the *EV31* cultures in the dark and at HL as well, at which the photosynthetic subunits were diminished (Supporting Information: Figures 4 and 5).

The morphological changes were much less pronounced in TP medium, and increased lipid droplet formation was seen only in the NL and HL‐treated *PSBO* amiRNA lines (Figure 8b, Supporting Information: Figure 6), in which the photosynthetic apparatuses were partially degraded (Supporting Information: Figures 4 and 5).

### 3.4 PSBO down‐regulation impacts cell growth and cell cycle progression

**Figure 9:**
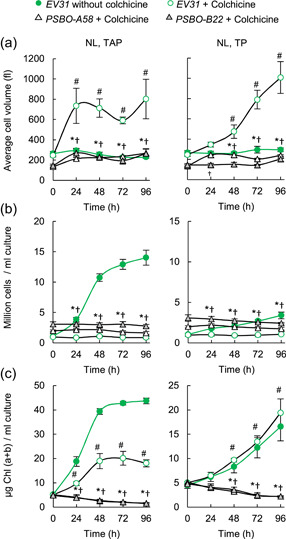
The effects of colchicine treatment to arrest cell division following down‐regulation of *PSBO* via the nitrate‐inducible amiRNA in the presence (TAP medium) or absence of acetate (TP medium) at normal light (100 µmol photons m^−2^ s^−1^, NL). (a) Changes in cell size following induction. (b) Changes in cell density following induction. (c) Changes in Chl (a + b) content following induction. Values are means ± SE of three biological replicates. Statistical significance levels are presented relative to the *EV31* (+Colchicine) strain (at each individual time‐point) as *p* < 0.05, *PSBO‐A58, †*PSBO‐B22*. For comparison with the *EV31* (−Colchicine, 0 h) sample, statistical significance levels are presented as #*p* < 0.05. amiRNA, artificial microRNA; Chl, chlorophyll; SE, standard errors; TAP, Tris‐acetate‐phosphate. [Color figure can be viewed at wileyonlinelibrary.com]

To investigate whether cell expansion is also affected by the down‐regulation of PSBO, cultures were treated with colchicine. Colchicine treatment of algal cells results in the arrest of cell division and the formation of giant cells (Walne et al., 1966). This is due to the interaction of colchicine with microtubules during mitosis, which in turn prevents the separation of chromosomes, thus resulting in polyploidy. As expected, colchicine treatment of the *EV31* strain increased cell size three‐ to fourfold and cell division stopped (Figure 9a,b), whereas Chl (a + b) content continued increasing to some extent (Figure 9c). Upon colchicine treatment and induction of the *PSBO* amiRNA lines at NL, the cell size remained largely unaltered (Figure 9a), suggesting that upon the down‐regulation of *PSBO*, cell expansion is inhibited, and consequently, the cells are unable to reach the critical size necessary for division.

**Figure 10:**
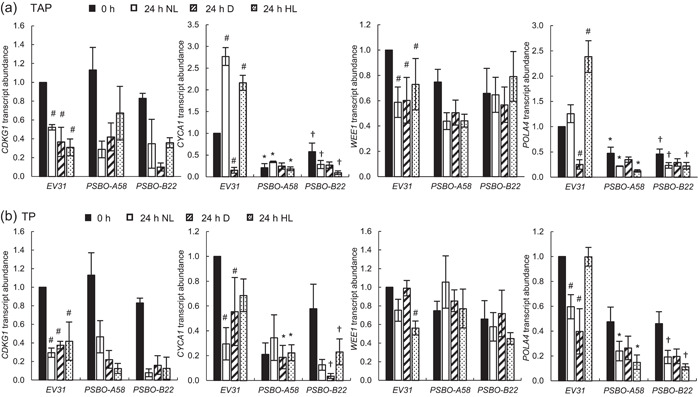
Transcript abundance of key cell division genes (*CDKG1, CYCA1, WEE1, POLA4*) 24 h after down‐regulation of PSBO via the nitrate‐inducible amiRNA approach at normal light (100 µmol photons m^−2^ s^−1^, NL), in the dark (D) and at high light (530 µmol photons m^−2^ s^−1^, HL). (a) Transcript abundance in the presence of acetate (TAP medium). (b) Transcript abundance in the absence of acetate (TP medium). Values are means ± SE of three biological replicates. Statistical significance levels are presented relative to the *EV31* strain (at each individual time‐point) as *p* < 0.05, *PSBO‐A58, †*PSBO‐B22*. For comparison with the (0 h) *EV31* sample, statistical significance levels are presented as #*p* < 0.05. amiRNA, artificial microRNA; SE, standard errors; TAP, Tris‐acetate‐phosphate.

To gain information on the cell division machinery during the induction of our *PSBO* amiRNA transformants, we examined the relative transcript levels of marker genes of cell division. *CDKG1* (Cre06.g271100) acts as a determinant of daughter cell size (Y. Li et al., 2016). Figure 10 shows that its expression was moderately decreased in all strains and all conditions when the cultures were transferred from ammonium to nitrate‐containing media (cf. 0 vs. 24 h NL), but there was no specific decrease in its expression in the *PSBO* amiRNA lines.

*CYCA1* (Cre03.g207900) is required to promote DNA replication and for efficient initiation of cytokinesis by mediating biochemical activation of CDKA1 (Atkins & Cross, 2018). Its expression was strongly downregulated in both *PSBO* amiRNA lines in all conditions, and even in the non‐induced cultures, there was a significant decrease in its abundance (Figure 10) that may be related to the inherent leakiness of nitrate‐inducible amiRNA expression (see Figure 1). The expression of *CYCA1* also decreased in the *EV31* strain when subjected to darkness in TAP medium. In TP medium, the differences between the *PSBO* amiRNA lines and the *EV31* control were similar but less pronounced (Figure 10b).

CDK/cyclin complexes are negatively regulated by WEE1 kinases that phosphorylate a conserved tyrosine residue of the CDK subunit. This negative regulation is necessary for the proper timing of mitosis (Gould & Nurse, 1989; Jin et al., 1996). *C. reinhardtii* has a single WEE1 ortholog encoded by the *WEE1* gene (Cre07.g355250), whose expression is up‐regulated during S/M phase, but not detectable at earlier stages (Bisova et al., 2005). We found that its transcript level is not particularly affected by the induction of the *PSBO* amiRNA construct, nor by the light conditions or the presence of acetate (Figure 10).

*POLA4* (Cre07.g312350) encodes the primase subunit DNA polymerase alpha. It was shown earlier by Kabeya and Miyagishima (2013) that, upon inhibition of photosynthetic electron transport, chloroplast DNA replication is diminished and the expression of nuclear‐encoded genes also decreases. DNA replication also appears to be regulated by the redox state of the chloroplast (Kabeya & Miyagishima, 2013). In agreement with this, we observed a decrease in *POLA4* transcript abundance upon induction of the *PSBO* amiRNA construct in all strains. In the non‐induced *PSBO* amiRNA lines, there was also a significant decrease in *POLA4* transcript abundance (Figure 10), probably related to the leakiness of the construct. The transcript abundance of *POLA4* was also low in the dark‐treated *EV31* cultures (Figure 10).

Together, the above‐described data suggest that, upon down‐regulation of *PSBO*, DNA synthesis is downregulated and cell division is halted.

### 3.5 The diminishment of PSBO protein level enhanced upon CO_2_− supply

**Figure 11:**
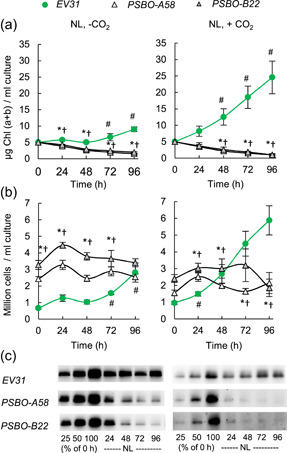
The effects of CO_2_ supplementation upon down‐regulation of *PSBO* via the nitrate‐inducible amiRNA in TP medium at 100 µmol photons m^−2^ s^−1^ (NL). (a) Changes in Chl (a + b) content following induction. (b) Changes in cell density content following induction. (c) Representative immunoblots to monitor the loss of PSBO in the three genotypes in nitrate‐containing TAP medium. The samples were loaded on equal cell numbers. Values are means ± SE of three biological replicates. Statistical significance levels are presented relative to the *EV31* strain (at each individual time‐point) as *p* < 0.05, *PSBO‐A58, †*PSBO‐B22*. For comparison with the (0 h) *EV31* sample, statistical significance levels are presented as #*p* < 0.05. amiRNA, artificial microRNA; Chl, chlorophyll; SE, standard errors; TAP, Tris‐acetate‐phosphate. [Color figure can be viewed at wileyonlinelibrary.com]

The above data indicate that the rate of PSBO diminishment is strongly dependent on the presence of acetate. To test whether this can be linked to carbon availability or specifically to acetate, cultures were grown in a photobioreactor in TP medium with or without 1% CO_2_ supplementation. There was a steady increase in Chl (a + b) content and cell number in the *EV31* strain upon CO_2_ supply, whereas, in the *PSBO* amiRNA lines, cell proliferation could not be observed upon induction (Figure 11a,b).

Upon CO_2_ supply, the PSBO content rapidly diminished, and without CO_2_ supply, the protein persisted longer (Figure 11c). These data show that when CO_2_ assimilation is active, the rate of PSBO degradation becomes faster.

## 4 DISCUSSION

### 4.1 The lifetime of PSBO in *C. reinhardtii* is dependent on light and carbon availability

PSBO is essential for oxygen evolution in photosynthetic organisms, including green algae. It stabilizes the Mn‐cluster and regulates the access of Ca^2+^, Cl^−^ as well as water and proton removal from the Mn‐cluster (Guskov et al., 2009; Ho & Styring, 2008; Loll et al., 2005; Offenbacher et al., 2013; Vinyard & Brudvig, 2017). In vitro studies have suggested that PSBO is a relatively stable protein (Hashimoto et al., 1996), in spite of its involvement in the energetically highly demanding water oxidation reaction. On the other hand, the Mn‐cluster may be prone to photoinhibition due to the absorption of visible light, which may affect the integrity of PSBO as well (Murata et al., 2007; Zavafer et al., 2015; Zavafer & Mancilla, 2021).

To our knowledge, the dependence of the PSBO lifetime on environmental conditions has not been carefully assessed in green algae. We have chosen *C. reinhardtii* as a model organism, since the stability of its OEC is particularly relevant for photosynthesis‐based biotechnological applications, such as hydrogen production (e.g., Nagy et al., 2021) and the production of various high‐value compounds (Mehariya et al., 2021). To study the dependence of PSBO lifetime on environmental conditions and the consequences of its down‐regulation, we decided to generate inducible amiRNA lines targeting the CDS (in the *PSBO‐A58* strain) or the 3'UTR (*PSBO‐B22* strain) of *PSBO* mRNA. Our nitrate‐inducible *PSBO* amiRNA transformants grew normally under non‐inducing conditions and their photosynthetic apparatus performed similarly to that of the control strains.

We observed a remarkable diminishment in *PSBO* mRNA level upon induction (Figure 4), and additionally, the amiRNA approach can act at the translational level in *C. reinhardtii* (Vidal‐Meireles et al., 2017). Therefore, it is very likely that the synthesis of new PSBO proteins was prevented to a large extent, although it is possible that a minor amount of PSBO was still produced in the *PSBO* amiRNA lines after induction. For this reason, we used the time course of PSBO diminishment as a qualitative descriptor of PSBO lifetime. A similar approach was used earlier in tobacco plants to obtain information about the lifetime of cytochrome b_6_f complex subunits (Hojka et al., 2014).

Upon induction of amiRNA expression, we observed a halt of cell division, a light‐dependent Chl (a + b) content loss, and a diminishment of PSBO level on a cell number basis. At NL and photomixotrophic conditions, the cellular PSBO content decreased by about 75% in 24 h and this loss was remarkably accelerated when the cultures were placed in HL, and slowed down when the cells were kept in the dark (Figure 3). The arrest of cell division infers that the induction of the *PSBO* amiRNA construct did not simply decrease the cellular PSBO pool by blocking de novo synthesis and limiting its supply to the daughter cells. The absence of such a dilution effect and the gradual diminishment of cellular PSBO content mean that PSBO has a significant, and light intensity‐dependent turnover.

We also found that, unexpectedly, in the absence of acetate, under photoautotrophic conditions (i.e., in TP medium), the cellular PSBO content was substantially more stable in comparison with acetate‐supplied cultures (Figure 3). Excess acetate has been shown to remarkably reduce photosynthetic carbon gain and oxygen evolution but without affecting the integrity of PSII (Heifetz et al., 2000). On the other hand, acetate also diminishes the yield of singlet oxygen production in *C. reinhardtii* (Roach et al., 2013). Thus, it is unlikely that acetate itself had a damaging effect on PSBO. Accordingly, when cultures were supplemented with 1% CO_2_, the lifetime of PSBO also decreased relative to CO_2_‐limited cultures in TP medium (Figure 11).

These data suggest that the lifetime of PSBO in *C. reinhardtii* is largely dependent on carbon availability. We hypothesize that this is related to the fact that acetate and CO_2_‐deprived cultures are metabolically less active and divide only slowly. It has been shown in a wide range of species, including yeast and mammals, that lifespan can be prolonged by reducing nutrient intake (López‐Otín et al., 2016; Sampaio‐Marques et al., 2019), which may be due to reduced metabolic activity and the concomitantly reduced ROS production (e.g., Munro & Pamenter, 2019); however, the role of ROS was challenged (e.g., Gladyshev et al., 2014) and the exact mechanism of action remains to be explored.

Recent results obtained on *C. reinhardtii* suggest that this so‐called caloric restriction concept may apply to microalgae as well. Zamzam et al. (2022) demonstrated that high levels of acetate and high starch levels substantially decrease longevity. In agreement with this, we observed that on a timescale of days, the F_V_/F_M_ parameter of control (*EV31*) cultures decreased more substantially in TAP medium than in TP medium (Figure 5), and in addition, the quantities of the tested photosynthetic subunits moderately decreased in TAP medium both at HL and in the dark, whereas in TP the subunits were stable (Figures 6 and 7). This suggests that, upon HL stress and when the photosynthetic apparatus does not need to remain functional (e.g., in the dark), ample carbon availability leads to diminished photosynthetic activity and to a reduced lifetime of photosynthetic complexes.

Zamzam et al. (2022) hypothesized that excess carbon availability causes an overreduction of the photosynthetic electron transport chain, and in this way, increased ROS production. The PQ‐pool may become reduced due to increased chlororespiration (e.g., Cardol et al., 2003) and this may seem a plausible signal participating in the regulation of the lifespan of photosynthetic complexes and that of the entire cell. On the other hand, as mentioned earlier, acetate rather decreases than increases ROS production in *C. reinhardtii* cultures (Roach et al., 2013). Thus the mechanism by which acetate and in general, carbon availability modifies the lifetime of all photosynthetic complexes, warrants further investigations.

In this study, we provide direct experimental evidence that reduced carbon availability expands the lifetime of the major OEC subunit, PSBO. At this stage, we cannot generalize this finding to other photosynthetic subunits, but it seems likely that PSBO is not unique in this respect, and the down‐regulation of other essential photosynthetic subunits could occur in a similar manner.

### 4.2 Down‐regulation of PSBO results in a complete disassembly of the photosynthetic apparatus

PSBO is required for the stable accumulation of PSII reaction centres in plants (Bricker & Frankel, 2011; Murakami et al., 2002). On the other hand, it is somewhat uncertain to what extent PSBO is required for PSII stability in green algae. The nitrate‐inducible amiRNA approach offers an appropriate tool to assess the consequences of downregulating *PSBO* expression in mature PSII complexes.

We found that upon the induction of the *PSBO* amiRNA construct in TAP medium, the amount of PSBO rapidly decreases, leading to PSII reaction centre inactivation, as reflected by complete loss of oxygen evolution, and a decrease in the F_V_/F_M_ value (Figure 5). The diminishment of cellular PSBO content also entails a strong reduction in the amounts of various photosynthetic subunits (Figure 6). In addition, accumulation of lipid droplets and altered chloroplast ultrastructure were observed, and the cultures finally bleached in the light (Figures 2 and 8). Thus, our data confirm that, in addition to its requirement for PSII assembly, PSBO is essential for PSII maintenance in green algae. This conclusion is in agreement with earlier studies on the FuD44 *C. reinhardtii* mutant that constitutively lacks PSBO (Mayfield et al., 1989; Pigolev et al., 2009; De Vitry et al., 1989) and the TSP4 temperature‐sensitive mutant (Bayro‐Kaiser & Nelson, 2020). The FuD44 mutant is unable to form active PSII units when grown in light, but a small amount of photochemically active reaction centres accumulate in the dark, without the capacity to evolve oxygen (Pigolev et al., 2009, 2012). The TSP4 mutant was characterized by a strong diminishment of the F_V_/F_M_ value and the losses of PSBO, PsbA and PetA at a moderately high temperature at which PSBO is highly unstable (Bayro‐Kaiser & Nelson, 2020).

The fact that the entire photosynthetic apparatus was degraded upon the loss of PSBO, indicate that a general cellular response was triggered, involving oxidative stress and possibly autophagy (Heredia‐Martínez et al., 2018; Meagher et al., 2021). The most likely scenario is that upon loss of PSBO, the OEC becomes inactivated, thereby electron transfer to Tyr_Z_^+^ and P680^+^ is interrupted, and highly oxidizing species accumulate, damaging the photosynthetic apparatus. This so‐called ‘donor‐side induced photoinhibition’ process occurs on the timescale of minutes and was first described for PSII reaction centres the OECs of which had been inactivated chemically (Blubaugh & Cheniae, 1990; Callahan et al., 1986; Chen et al., 1995; Jegerschöld & Styring, 1996). ‘Donor‐side induced photoinhibition’ occurs in heat‐treated leaves as well. It is initiated by the release of PSBO (and possibly other OEC subunits) and loss of Ca^2+^ and Cl^−^ from the Mn‐cluster, leading to the loss of OEC activity. Consequently, PSII reaction centres are inactivated within minutes, and they are degraded on the timescale of a few hours (Tóth et al., 2011). This is in agreement with the observation made in this study that at 24 h after *PSBO* amiRNA induction, the amounts of various photosynthetic subunits are strongly diminished (Figure 6).

The induction of the *PSBO* amiRNA construct in TAP medium led to the loss of PSBO also in the dark (Figure 3), and this resulted in the diminishment of several photosynthetic subunits, including PsbA, PsbP, PetA, RbcL and CP47 (Figure 6). These results show that the loss of PSBO triggers a regulated degradation of photosynthetic complexes, which is independent of the presence of light and, therefore, probably independent of oxidative stress. Thus, hypothetically, PSBO may also play a specific role in maintaining the homoeostasis of the photosynthetic apparatus. Upon down‐regulation of PSBO, cell division and expansion ceased both in TAP and TP media (Figures 2 and 9), along with a decrease in the expression levels of *CYCA1* and *POLA4* (Figure 10), encoding proteins participating in DNA replication. These findings can be partly explained by the regulatory effect of photosynthetic electron transport and the redox state of the chloroplast on DNA replication (Kabeya & Miyagishima, 2013; Ohbayashi et al., 2013), but the exact mechanism by which PSBO contributes to cellular homoeostasis, certainly warrants further investigations.

## 5 CONCLUSIONS

In microalgae, the adjustment of the photosynthetic complex stoichiometry may occur through cell division that is coupled with the tuning of gene expression (Davis et al., 2013; Meagher et al., 2021). In this scenario, the turnover of photosynthetic subunits is of limited importance. However, the regulation of cell division cannot explain how algae can cope with varying light conditions under nutrient‐limiting conditions when cell division is limited. We have demonstrated that in contrast to previous notions, PSBO has a significant, light, and carbon‐supply‐dependent turnover and its quantity is not regulated only through cell division.

Determining the lifetime of specific photosynthetic subunits is of high importance both from the point of view of deciphering the mechanisms of photosynthesis and its regulation, and for the bio‐industrial exploitation of green algae. In the bio‐industry, a major endeavour is to maintain algal cultures and their productivity for an extended period of time, due to the high costs of establishing new algal cultures. Moreover, biomass accumulation is restricted for example, in biofilm culturing systems (Leino et al., 2012; Vajravel et al., 2020) and in batch hydrogen production systems with restricted carbon assimilation (Kosourov et al., 2018; Nagy et al., 2021). In this work, we demonstrated the prominent role of PSBO in sustaining the homoeostasis of the photosynthetic apparatus. We have also found that its lifetime is prolonged in moderate light and darkness, and in the absence of ample carbon supply.

## Supporting information

Supplementary information.

## Data Availability

The data that support the findings of this study are available from the corresponding author upon reasonable request. All new created data are contained within this article and in its supplementary material of this article. All biological materials generated for this work will be available upon request.
