# Supplementary material for: The lifetime of the oxygen‐evolving complex subunit PSBO depends on light intensity and carbon availability in Chlamydomonas
Source: Plant Cell Environ. 2022 Nov 17;46(2):422–39. doi: 10.1111/pce.14481 (PMC10100022; doi:10.1111/pce.14481)
Supplement: Supplementary file 1 — Supplementary information. [file PCE-46-422-s001.pdf]

Name	Sequence
PSBO_CDS1_amiRNA_fw	ctagtAGGGTAGCGAGACTGACTTTAtctcgctgatcggcaccatgggggtggtggtgatcagcgctaTAAACTCAGTCTCGCTACCCTg
PSBO_CDS1_amiRNA_rev	ctagcAGGGTAGCGAGACTGAGTTTAtagcgctgatcaccaccacccccatggtgccgatcagcgagaTAAAGTCAGTCTCGCTACCCTa
PSBO_3'UTR_amiRNA_fw	ctagtGAGGAACTCTAGCGGGAGTAAtctcgctgatcggcaccatgggggtggtggtgatcagcgctaTTACACCCGCTAGAGTTCCTCg
PSBO_3'UTR_amiRNA_rev	ctagcGAGGAACTCTAGCGGGTGTAAtagcgctgatcaccaccacccccatggtgccgatcagcgagaTTACTCCCGCTAGAGTTCCTCa
pNIT1fw	CGCTGCATTGTGGCTTGAAGG
amiRNA-Hsf1rev	GCTAGCGCGCCTTGGTTTCCT
PSBO_transcript_qPCR_fw	AACACTTCTGGAGGAGGAGGA
PSBO_transcript_qPCR_rev	TTGCCTACCCTTCCAATCCC
Pulsed_PSBO-A_RT	GTGCAGGGTCCGAGGTCAGAGCCACCTGGGCAATTTTTTTTTTTATGGGA
PSBO-A_amiRNA_fw	GCGGCGTAAACTCAGTCTCGC
Pulsed_PSBO-B_RT	GTGCAGGGTCCGAGGTCAGAGCCACCTGGGCAATTTTTTTTTTTAAGGAG
PSBO-B_amiRNA_fw	GCGGCGTTACACCCGCTAGAG
Pulsed_U4-snoRNA_RT	GTGCAGGGTCCGAGGTCAGAGCCACCTGGGCAATTTTTTTTTTTATTTCTC
U4-snoRNA_fw	CGGCGCAAAAGGCCCGACAGAAAT
Universal-qPCR_rev	CAGTGCAGGGTCCGAGGT

Supplementary Table 1 - Sequences of the amiRNA targeting the coding region (CDS) and the 3'UTR of PSBO, oligonucleotide primers for screening the correct amiRNA clones, RT primers used in pulsed PCR incubation, and primers used in real-time qPCR analysis for detection of the PSBO transcript and PSBO amiRNA, respectively.

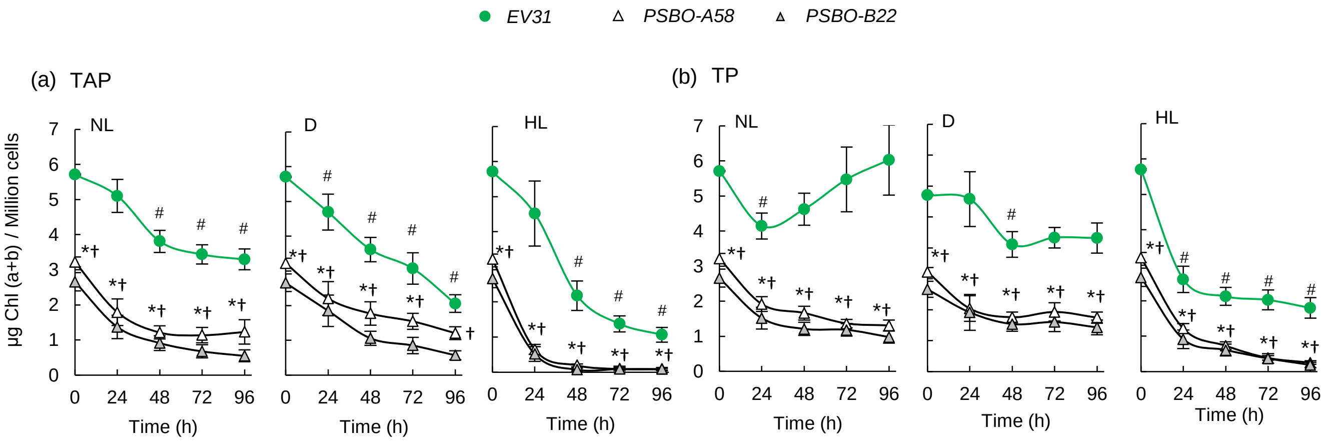

Supplementary Figure 1 – The effects of downregulating *PSBO* via the nitrate-inducible amiRNA approach on the cellular Chl(a+b) content at normal light (100 µmol photons $m^{-2}s^{-1}$, NL), in the dark (D), and at high light (530 µmol photons $m^{-2}s^{-1}$, HL). (a) Changes in Chl(a+b)/million cells content following induction in TAP medium. **(b)** Changes in Chl(a+b)/million cells content following induction in TP medium. Values are means ± SE of four biological replicates. Statistical significance levels are presented relative to the *EV31* strain (at each individual time-point) as $p<0.05$, * *PSBO-A58*, † *PSBO-B22*. For comparison with the (0 h) *EV31* sample, statistical significance levels are presented as # $p<0.05$.

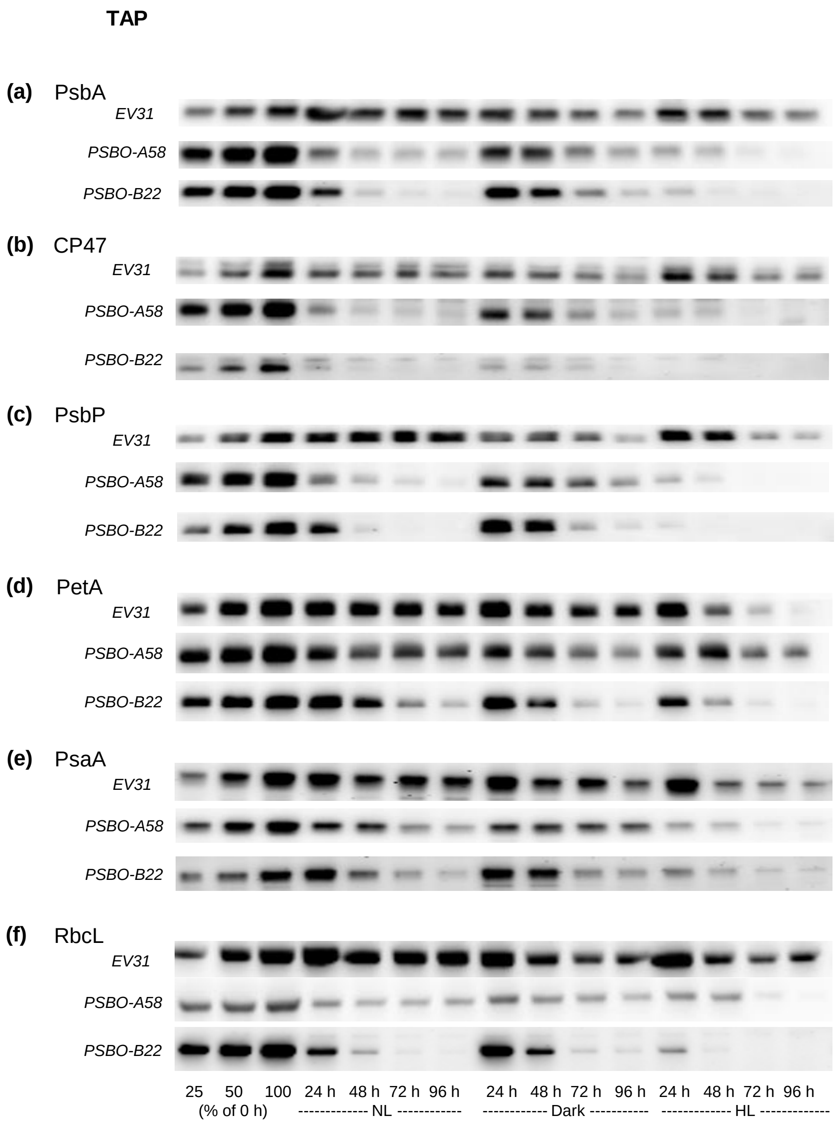

Supplementary Figure 2 – Representative immunoblots of certain photosynthetic subunits following downregulating *PSBO* via the nitrate-inducible amiRNA approach in the presence of acetate (TAP medium) at normal light (100 µmol photons m^{-2} s^{-1}, NL), in the dark (D), and at high light (530 µmol photons m^{-2} s^{-1}, HL). (**a**) PsbA. (**b**) CP47. (**c**) PsbP. (**d**) PetA. (**e**) PsaA. (**f**) RbcL. Samples were loaded based on equal cell numbers. The 0 h samples represent the uninduced control, the 25%, 50%, and 100% dilution series are for the approximate quantitation of the proteins.

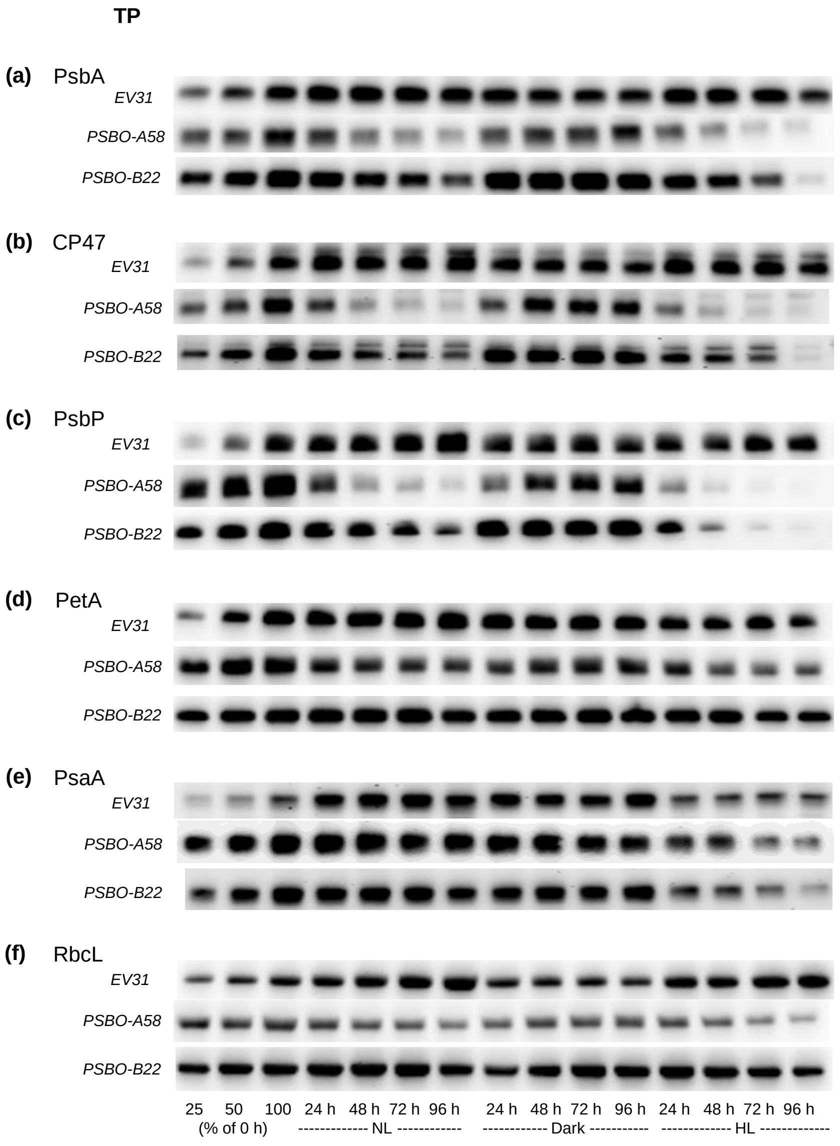

Supplementary Figure 3 – Representative immunoblots of certain photosynthetic subunits following downregulating *PSBO* via the nitrate-inducible amiRNA approach in the absence of acetate (TP medium) at normal light (100 µmol photons m^{-2} s^{-1}, NL), in the dark (D), and at high light (530 µmol photons m^{-2} s^{-1}, HL). (**a**) PsbA. (**b**) CP47. (**c**) PsbP. (**d**) PetA. (**e**) PsaA. (**f**) RbcL. Samples were loaded based on equal cell numbers. The 0 h samples represent the uninduced control, the 25%, 50%, and 100% dilution series are for the approximate quantitation of the proteins.

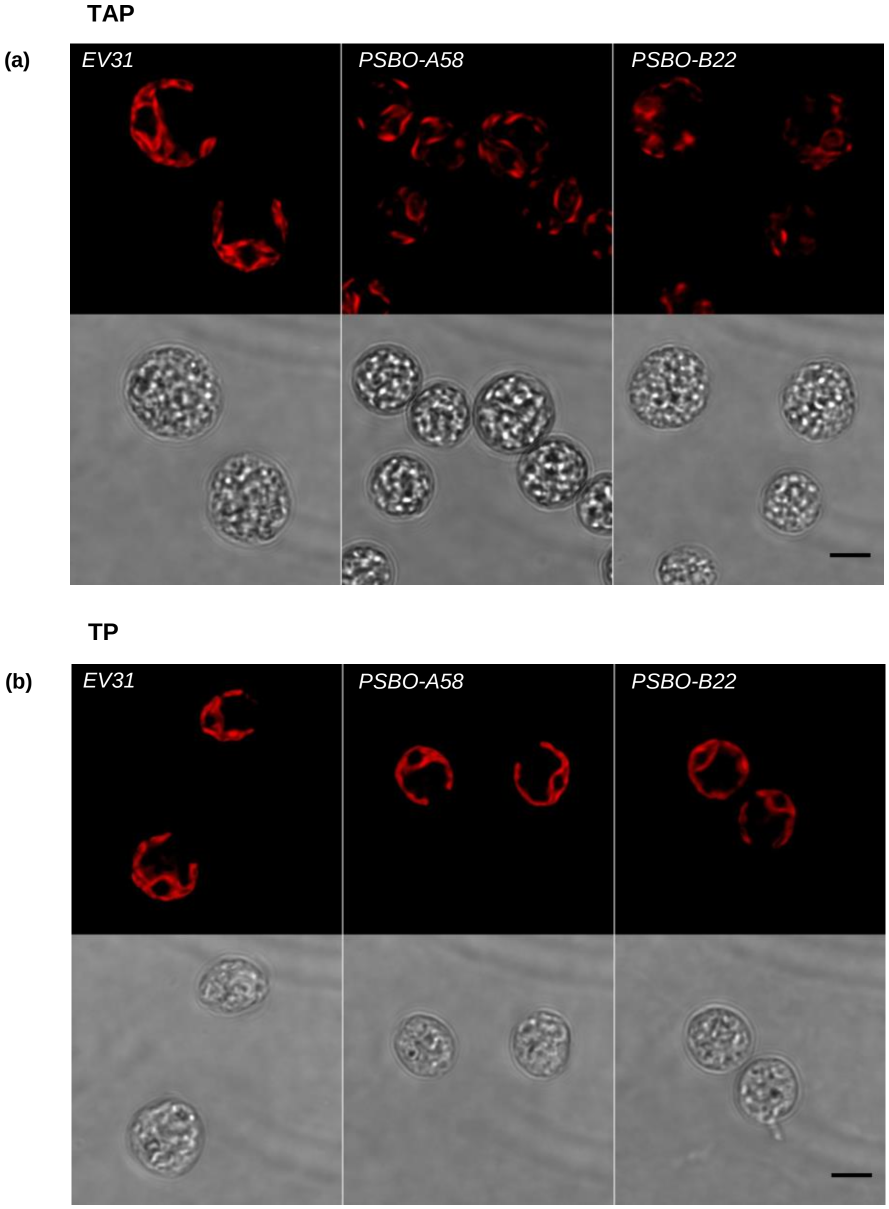

Supplementary Figure 4 – Confocal fluorescence microscope images at 48 h following downregulating PSBO via the nitrate-inducible amiRNA approach in the dark. Single plane chlorophyll auto-fluorescence (red) and their corresponding transmission images (black and white) are shown. (**a**) In the presence of acetate (TAP). (**b**) In the absence of acetate (TP). Scale bar 5 µm.

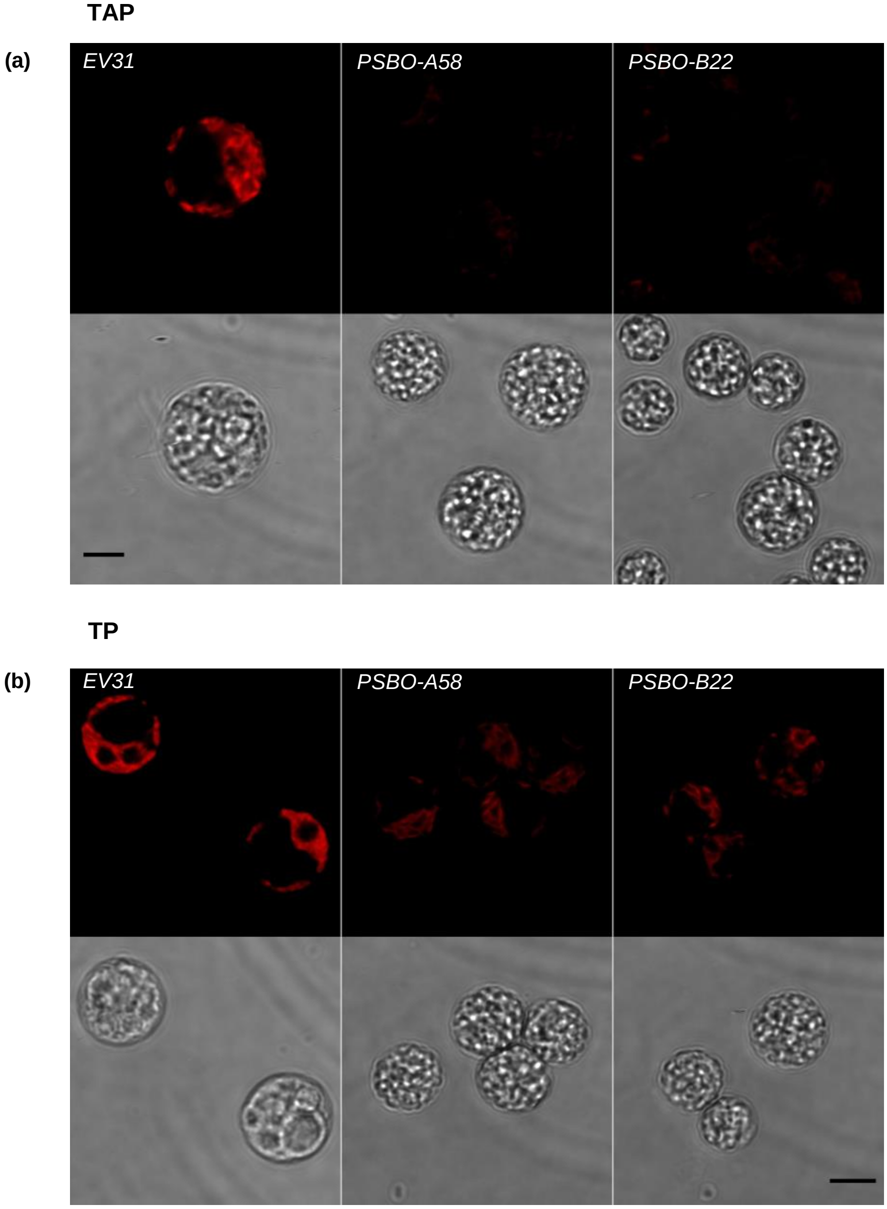

Supplementary Figure 5 – Confocal fluorescence microscope images at 48 h following downregulating PSBO via the nitrate-inducible amiRNA approach at high light (530 µmol photons m^{-2} s^{-1}). Single plane chlorophyll auto-fluorescence (red) and their corresponding transmission images (black and white) are shown. (**a**) In the presence of acetate (TAP). (**b**) In the absence of acetate (TP). Scale bar 5 µm.

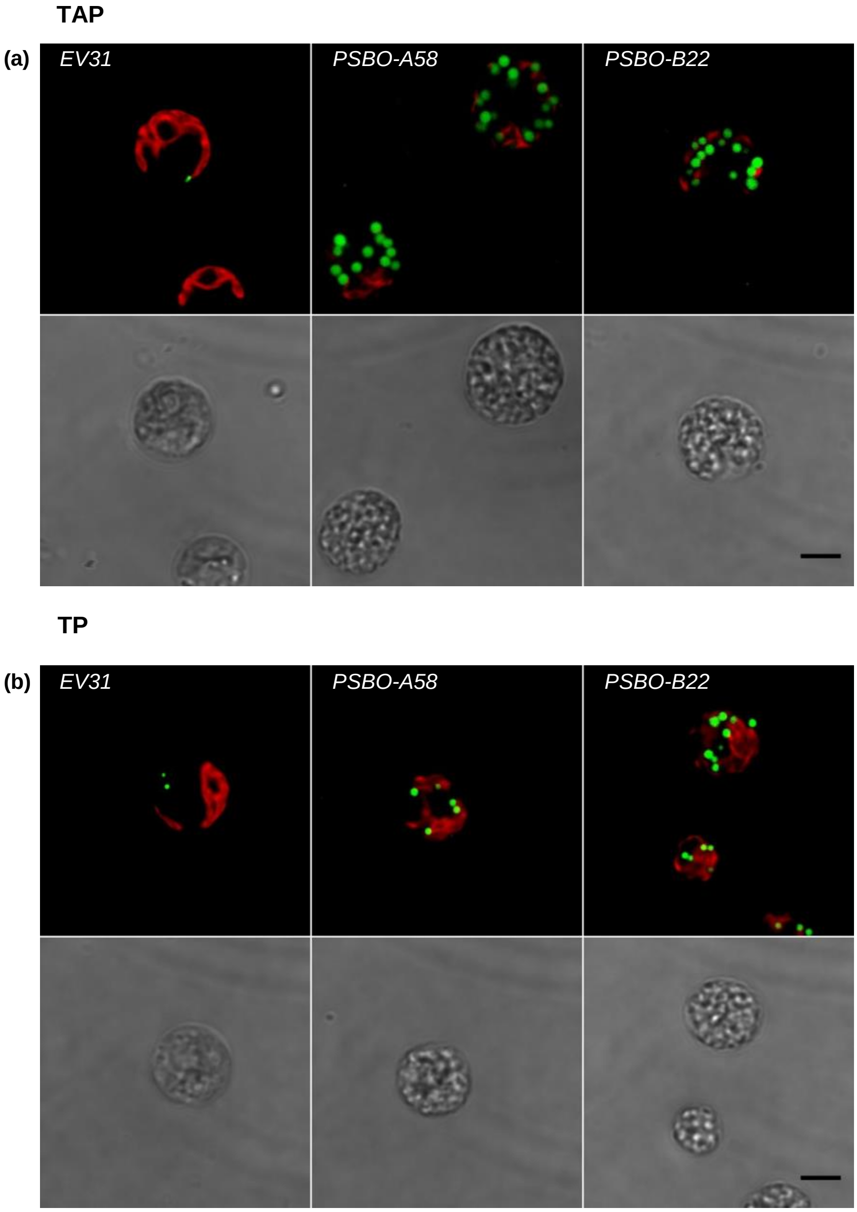

Supplementary Figure 6 – Confocal fluorescence microscope images of Nile Red stained lipid droplets at 48 h following downregulating PSBO via the nitrate-inducible amiRNA approach at normal light (100 µmol photons m^{-2} s^{-1}) . Single plane transmission images (black and white) and their corresponding chlorophyll auto-fluorescence (red) and Nile Red stained lipid droplets (green) merge images are shown. (**a**) In the presence of acetate (TAP). (**b**) In the absence of acetate (TP). Scale bar 5 µm.
